# Effect of topical bromfenac on intraretinal cystoid lesion in simultaneous cataract and idiopathic epiretinal membrane surgery

**DOI:** 10.1186/s12886-024-03380-2

**Published:** 2024-03-15

**Authors:** EunAh Kim, Han Jo Kwon, Sung Who Park, Iksoo Byon

**Affiliations:** 1 Department of Ophthalmology, Inje University Haeundae Paik Hospital, Busan, South Korea; 2 Department of Ophthalmology, Biomedical Research Institute, Pusan National University Hospital, Busan, South Korea; 3 Pusan National University School of Medicine, Yangsan, South Korea

**Keywords:** Macular edema, Idiopathic epiretinal membrane, Intraretinal cystic lesion, Microvascular leakage, Topical NSAIDs

## Abstract

**Purpose:**

To investigate the effect of topical nonsteroidal anti-inflammatory drugs (NSAIDs,) bromfenac on the intraretinal cystic lesions (IRC) when performing simultaneous cataract and idiopathic epiretinal membrane (iERM) surgery.

**Methods:**

This study included patients with iERM who had been followed up for 6 months after vitrectomy, membrane removal, and concurrent cataract surgery. Eyes were treated with topical bromfenac or not. The baseline fluorescein angiography (FA) was obtained to assess the microvascular leakage (ML). Structural changes of macula, including IRC and central macular thickness (CMT) were assessed using optical coherence tomography (OCT). The main outcome measures were changes in IRCs and best-corrected visual acuity (BCVA) regarding FA findings.

**Results:**

One hundred eighteen eyes were included. IRC and ML were observed in 51 eyes (43.2%) and 63 eyes (53.4%), respectively. The IRC did not show any association with the ML. Of total, 29 eyes (24.6%) were treated with topical bromfenac (Group A). Compared to Group B, topical bromfenac did not show beneficial effects in aspect of preventions for the newly developed IRC and treatment for pre-existed IRC. Whether the ML existed or not, topical bromfenac did not show any different effect on the changes in BCVA and IRC.

**Conclusion:**

When performing simultaneous cataract and ERM surgery, topical NSAIDs, bromfenac did not show beneficial effects on the preventions and treatment of IRC in both eyes with and without the ML.

## Introduction

The epiretinal membrane (ERM) is an amorphous membranous tissue on the innermost surface of the retina that induces deformity of the macular structure by thickening and wrinkling. Idiopathic ERM (iERM), which is most common, is associated with posterior vitreous detachment [1]. The iERM gradually progresses with proliferation and contraction and decreases the visual acuity over time. When patients with iERM complain of visual impairment, they are surgically treated with vitrectomy and membrane removal. Simultaneous cataract surgery is preferred in iERM surgery even if visually significant cataracts are not observed, based on the rapid development and progression of cataract in ageing patients.

Prolonged intraocular inflammation can sometimes induce cystoid macular edema (CME), which is a common cause of visual loss following uncomplicated cataract surgery in healthy eyes. Furthermore, Mylonas et al. [2] reported that postoperative CME was more frequently observed in patients with previous ERM surgery than in healthy controls. Regarding these, postoperative CME may be more affected by simultaneous cataract and ERM surgery. Interestingly, almost half of iERM cases already present CME or intraretinal cyst (IRC) at baseline [3–5]. It is reportedly a poor prognostic factor [4]. In addition, we reported that half of iERM eyes demonstrated the microvascular leakage (ML) on fluorescein angiography (FA) and showed advanced features and poor surgical outcomes [3]. Such eyes may be more vulnerable to extravascular leakage induced by an inflammatory reaction following combined cataract and ERM surgery.

Topical non-steroidal anti-inflammatory drugs (NSAIDs) can efficiently lower the incidence of CME after cataract surgery [6–8] Many studies consistently reported that topical NSAIDs reduced intravitreal level of prostaglandin E2 (PGE2) [9–11]. However, there was discrepancy about the effect of topical NSAID in the clinical setting [11, 12]. Moreover, a paucity of literature limits our understanding of the efficacy of topical NSAIDs in improving the surgical outcomes in the macular diseases. Herein, we investigated the effect of topical bromfenac on the macular edema (i.e., IRC) and visual acuity following vitrectomy, membrane removal, and simultaneous cataract surgery in patients with iERM subjected to FA at baseline.

## Materials and methods

### Participants

This retrospective cohort study included patients with iERM who simultaneously underwent vitrectomy, membrane removal, and cataract surgery at the vitreoretinal services of Pusan National University Hospital and Pusan National University Yangsan Hospital, South Korea, between July 2014 and July 2020. All patients were diagnosed based on the presence of a fibrous membrane on the macula detected using fundus photography (Canon CR-2 digital non-mydriatic retinal camera; Canon Inc., Tokyo, Japan) and swept-source OCT (Triton, Topcon Medical Systems, NJ). This study included patients who had intact ellipsoid zones of the photoreceptor layers and who had been followed up for at least 6 months postoperatively. Patients with discontinuous or absent ellipsoid zone lines, except for shadows from the retinal vessels and foldings in a volume scan of OCT, were excluded. We excluded patients with secondary ERM (denoted by a history of a retinal break with or without detachment, uveitis, intraocular tumour, or retinal vascular diseases), other intraocular diseases such as glaucoma and diabetic retinopathy, age-related macular degeneration, inherited retinal dystrophies, high myopia (spherical equivalent ≥ − 6.0 dioptre or axial length ≥ 26.5 mm), or a history of intraocular surgery. Additionally, patients with media opacities and cataracts interfering with visual acuity and detailed image analyses at baseline and those with intraoperative complications, including posterior capsule rupture, retinal breaks, and vitreous haemorrhage, were excluded.

This study was approved by the Institutional Review Board of the Pusan National University Hospital (approval no. 2209-014-119). Each patient was informed of the risks and benefits of the examinations and surgical treatment, and written informed consent was obtained from all patients. The study adhered to the tenets of the Declaration of Helsinki.

### Measurements

All eyes underwent comprehensive ophthalmic examinations, including best-corrected visual acuity (BCVA) assessment, slit-lamp examination, indirect ophthalmoscopy, fundus photography, and OCT at baseline and 6 months, as well as baseline FA. BCVA was measured using a Snellen chart and converted to the logarithm of the minimal angle of resolution (logMAR) for statistical analysis. Central macular thickness (CMT), IRC, and ectopic inner foveal layer (EIFL) were assessed using OCT. The scan protocol comprised a three-dimensional volumetric scan of 6 × 6 mm. The CMT was automatically calculated for a 1-mm foveal area using built-in software. The IRC was defined as the presence of cystic cavities in the sensory retina either with an elongated stellate pattern in the outer plexiform layer and Henle fibre layer or a rounded vertical cylindrical pattern in the inner nuclear layer [13]. The EIFL was defined as the presence of a continuous hypo- or hyper-reflective band extending from the inner nuclear layer and inner plexiform layer across the foveal region on OCT [14].. The ML was assessed from the venous to the late phase of FA [3].

### Surgical procedures and topical eyedrops treatments

Cataract surgery and vitrectomy were performed using the Constellation vitrectomy system (Alcon Laboratories, Inc. Fort Worth, TX) and a noncontact viewing system (Resight 700, Carl Zeiss Meditec, Jena, Germany). Cataract surgery was performed for all eyes through a 2.8-mm superior clear corneal incision before pars plana vitrectomy. A standard three-port 25-gauge vitrectomy was performed using a valved cannular system. The ERM was peeled tangentially using intraocular forceps after determining complete posterior vitreous detachment and vitreous removal. Internal limiting membrane (ILM) stained with 0.025% brilliant blue G solution (Sigma-Aldrich, St. Louis, MO) was peeled in 3–4 disc-diameter in all eyes. All the surgeries were performed by a single surgeon (I.B.) The eyes included in this study were divided into Group A and Group B based on the postoperative use or non-use of topical bromfenac (1.0 mg/mL of bromfenac sodium hydrate; Bronuck®, Taejoon Pharmaceutical, South Korea), respectively. Both groups used topical antibiotics and steroids four times a day for 2 weeks postoperatively. Group A additionally used bromfenac twice a day from 1 month to 6 months.

### Main outcome measures and statistical analysis

The main outcome measures included changes in the BCVA and OCT findings. The Shapiro-Wilk test was used to assess the normal distribution of the numeric variables. The independent numerical variables were analysed using t-tests. A paired t-test was used to analyse differences in the CMT. The Levene’s test was used to test the equal variance of the numeric variables. Mann–Whitney U test was used for comparing BCVA and independent non-numerical variables. The McNemar test was performed to analyse changes in the presence of IRC and EIFL. Statistical Product and Service Solutions, version 21.0 KR, for AU2 Windows (IBM) was used for the statistical analyses; *p*-values < 0.05 were considered statistically significant.

## Results

### Demographics

This study included 118 eyes of 118 patients. Topical bromfenac was prescribed for 29 eyes (Group A). The mean age was 66.6 ± 7.2 years (range: 51–87 years), and 42 patients (35.6%) were male. The baseline median BCVA was 0.40 (interquartile range: 0.30, 0.50), which improved to 0.20 (0.10, 0.20) postoperatively (*p* < 0.001). The mean (± standard deviation) CMT showed a significant reduction from 434.0 ± 82.9 μm to 350.8 ± 53.6 μm (*p* < 0.001). IRC was observed in 51 eyes (43.2%) at baseline and 41 eyes (34.7%) at 6 months. ML was observed in 63 eyes (53.4%) and EIFL in 72 eyes (61.0%) at baseline (Table 1). The IRC was present in 30/63 eyes (47.6%) in ML positive group and in 21/55 eyes (38.2%) in ML negative group, and the difference was not significant between two groups at baseline. The numbers of eyes with iERM stage 2, 3, and 4 were 7 (24.1%), 20 (69.0%), and 2 (6.9%) in Group A and 39 (43.8%), 47 (52.8%), and 3 (3.4%) in Group B, respectively, and there was no difference in the distribution between two groups.

**Table 1:** Patients’ characteristics

		Group A		Group B		*P*	
Age, mean (SD), years		66.3 (7.0)		66.7 (7.3)		0.790^a^	
Sex (female: male)		18: 11		58: 31		0.763^b^	
BCVA, median (IQR), LogMAR							
*Baseline*		0.40 (0.30, 0.50)		0.40 (0.30, 0.50)		0.534^b^	
*3 months*	*6 months*	0.20 (0.10, 0.30)	0.20 (0.00, 0.30)	0.20 (0.10, 0.40)	0.10 (0.10, 0.30)	0.731^b^	0.985^b^
*P*		< 0.001^c^	< 0.001^c^	< 0.001^c^	< 0.001^c^		
CMT, mean (SD), µm							
*Baseline*		439.3 (70.0)		432.2 (87.0)		0.657^a^	
*3 months*	*6 months*	376.6 (38.2)	370.7 (34.2)	360.0 (58.4)	344.4 (57.3)	0.156^a^	< 0.05^a^
*P*		< 0.001^d^	< 0.001^d^	< 0.001^d^	< 0.001^d^		
IRC, n, positive: negative							
*Baseline*		15: 14		36: 53		0.289^b^	
*3 months*	*6 months*	14: 15	12: 17	41: 48	29: 60	0.755^b^	0.395^b^
*P*		1.000^e^	0.508^e^	0.383 ^e^	0.281^e^		
EIFL, n, positive: negative							
*Baseline*		22: 7		50: 39		0.060^b^	
*3 months*	*6 months*	15: 14	13: 16	32: 57	32: 57	0.134^b^	0.395^b^
*P*		< 0.05 ^e^	< 0.01^e^	< 0.001^e^	< 0.001^e^		
ML, n, positive: negative -		18: 11		45: 44		0.283^b^	

*BCVA* best-corrected visual acuity, *CMT* central macular thickness, *EIFL* ectopic inner foveal layer, *IRC* intraretinal cystic lesions, *IQR* interquartile range, *ML* microvascular leakage

^a^t-test, ^b^Mann–Whitney U test, ^c^Wilcoxon signed-rank test, ^d^paired t-test, ^e^McNemar test

### Comparison of eyes treated with and without topical bromfenac

The BCVA significantly improved in both groups (from 0.40 [0.30, 0.50] to 0.20 [0.00, 0.30] in Group A and from 0.40 [0.30, 0.50] to 0.10 [0.10, 0.30] in Group B (*p* < 0.001 in both). The BCVA did not differ between two groups. The CMT also significantly improved in two groups (Group A; from 439.3 ± 70.0 to 370.7 ± 34.2 μm [*p* < 0.05]; Group B; from 432.2 ± 87.0 to 344.4 ± 57.3 μm [*p* < 0.001]). There was a greater reduction in the CMT in Group B than in Group A (*p* < 0.05).

Of the 51 eyes with IRC at baseline, 15 eyes (51.7%) belonged to Group A and 36 eyes (40.4%) to Group B. Both groups did not show a decrease in IRC postoperatively (Group A, 12 eyes [41.4%]; Group B, 29 eyes [32.6%] at 6 months). EIFL significantly decreased from 22 (75.9%) to 13 eyes (44.8%) (*p* < 0.05) in Group A and from 50 (56.2%) to 32 eyes (36.0%) in Group B, respectively (*p* < 0.001). Between two groups, there was no difference in the proportion of eyes with persistent, resolved and newly-developed IRC, and that with disappeared EIFL. Baseline ML also did not differ (Group A, 18 eyes [62.1%] vs. Group B, 45 eyes [50.6%]) (Table 1).

### Effect of topical bromfenac on the resolution and prevention of intraretinal cystic lesions

**Fig. 1:**
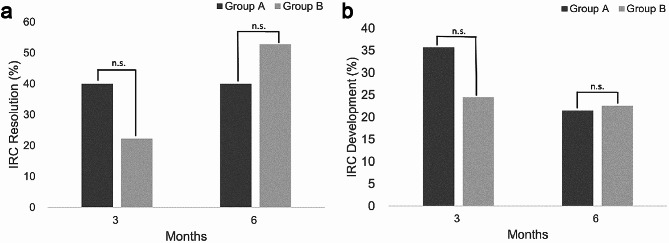
Changes in IRC after combined cataract and epiretinal membrane surgery. (**a**) In eyes with IRC, the resolution of IRCs was observed in 40.0% of group A and 22.2% of group B at 3 months, and 40.0% of group A and 52.8% of group B at 6 months, respectively. (**b**) In eyes without IRC, the development of new IRCs was in 35.7% of group A and 24.5% of group B at 3 months, and 21.4% of group A and 22.6% of group B at 6 months, respectively. There was no difference in the resolution and development of IRC between two groups through the follow-up visits. *IRC* intraretinal cystic lesions, *n.s*.: statistically not significant

In eyes with IRC, 6 eyes (40%) of Group A and 19 eyes (52.8%) of Group B presented resolution of IRC at 6 months. There was no difference in IRC resolution between two groups. Regarding eyes with IRC resolution, all 6 eyes in Group A achieved it at 3 months, but 8 eyes (42.1%) in Group B did (*p* < 0.05) (Fig. 1a). Of the 67 eyes without IRC at baseline (14 eyes in Group A and 53 eyes in Group B), newly developed IRC was in 15 eyes (22.4%). There was no difference in IRC development between two groups [3 eyes (21.4%) in Group A and 12 eyes (22.6%) in Group B]. Thus, topical bromfenac showed no relevance in the prevention of IRC development (Fig. 1b).

### Effects of topical bromfenac on surgical outcomes in eyes with and without microvascular leakage

**Fig. 2:**
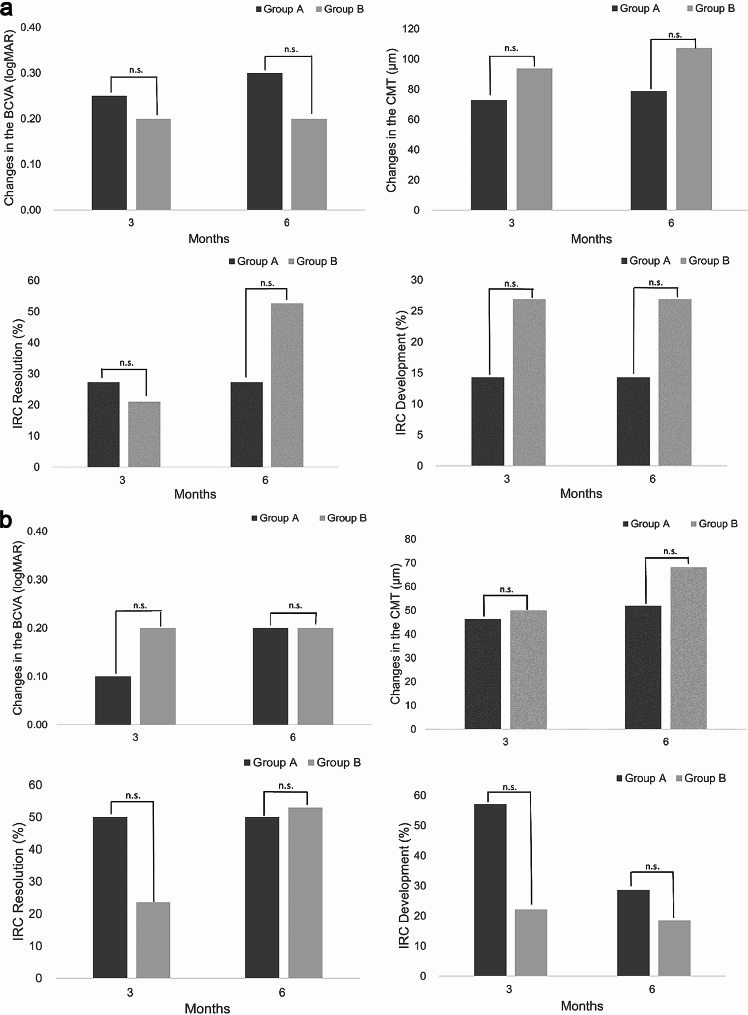
Comparison of eyes with and without microvascular leakage. (**a**) In eyes with ML, changes in the median BCVA and the mean CMT, and the resolution and development of IRC were not different between two groups through the follow-up period. (**b**) In eyes without ML, topical bromofenac did not provide any statistical difference in changes in the median BCVA and the mean CMT, and the resolution and development of IRC. *ML* microvascular leakage, *BCVA* best-corrected visual acuity, *CMT* central macular thickness, *IRC* intraretinal cystic lesions, *n.s*.: statistically not significant

**Fig. 3:**
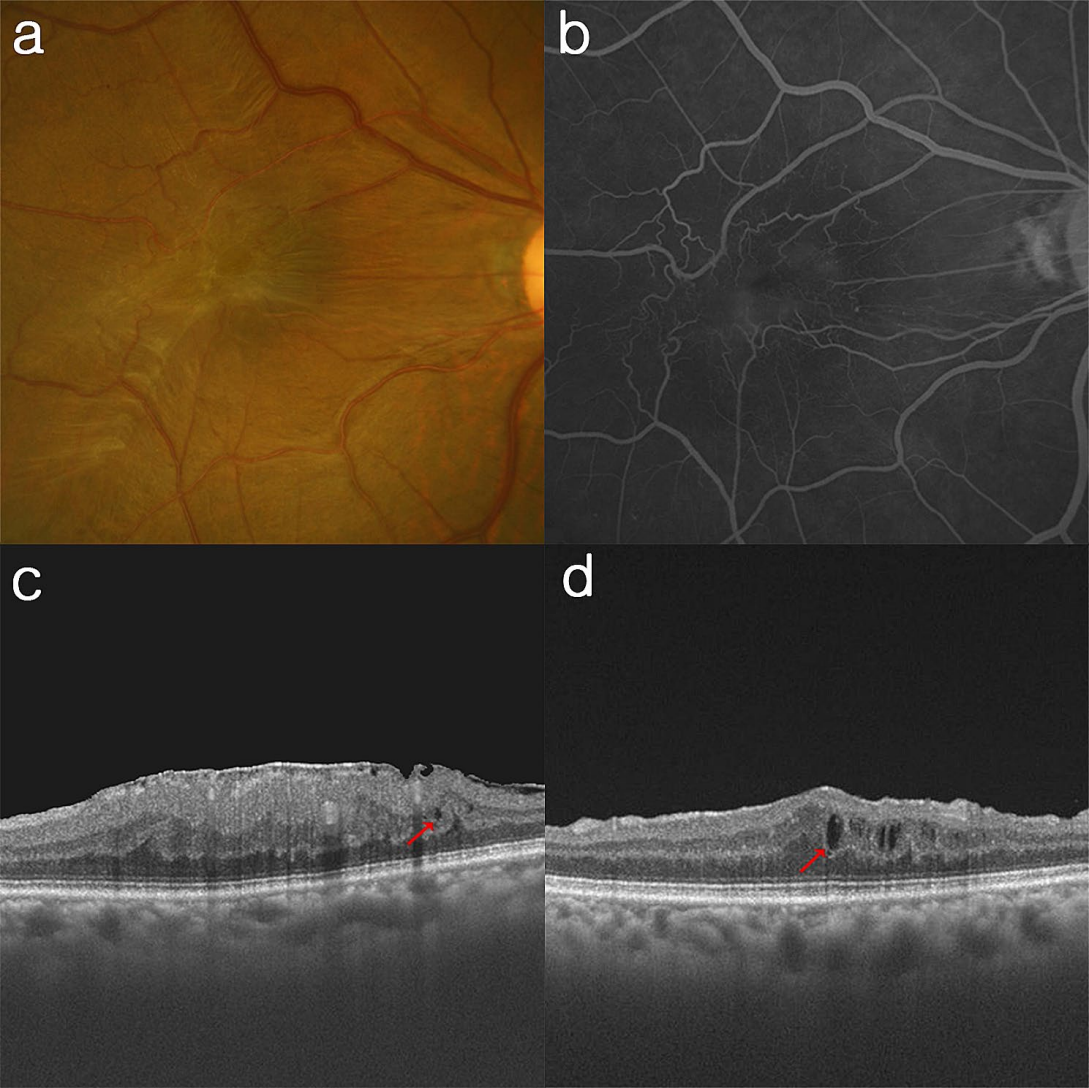
Representative case of a patient treated with topical bromfenac. (**a)** Fundus photography shows an opaque ERM with macular deformity. (**b)** FA presents microvascular leakage on macula. **(c)** A few IRCs (red arrow) are detected on the baseline OCT. (**d)** The IRCs (red arrow) increased after surgery despite of topical bromfenac use. The BCVA (from 20/200 to 20/200), and CMT (from 556 μm to 412 μm) showed limited improvement. *BCVA* best-corrected visual acuity, *CMT* central macular thickness, *ERM* epiretinal membrane, *FA* fluorescein angiography, *IRC* intraretinal cystic lesion, *OCT* optical coherence tomography

**Fig. 4:**
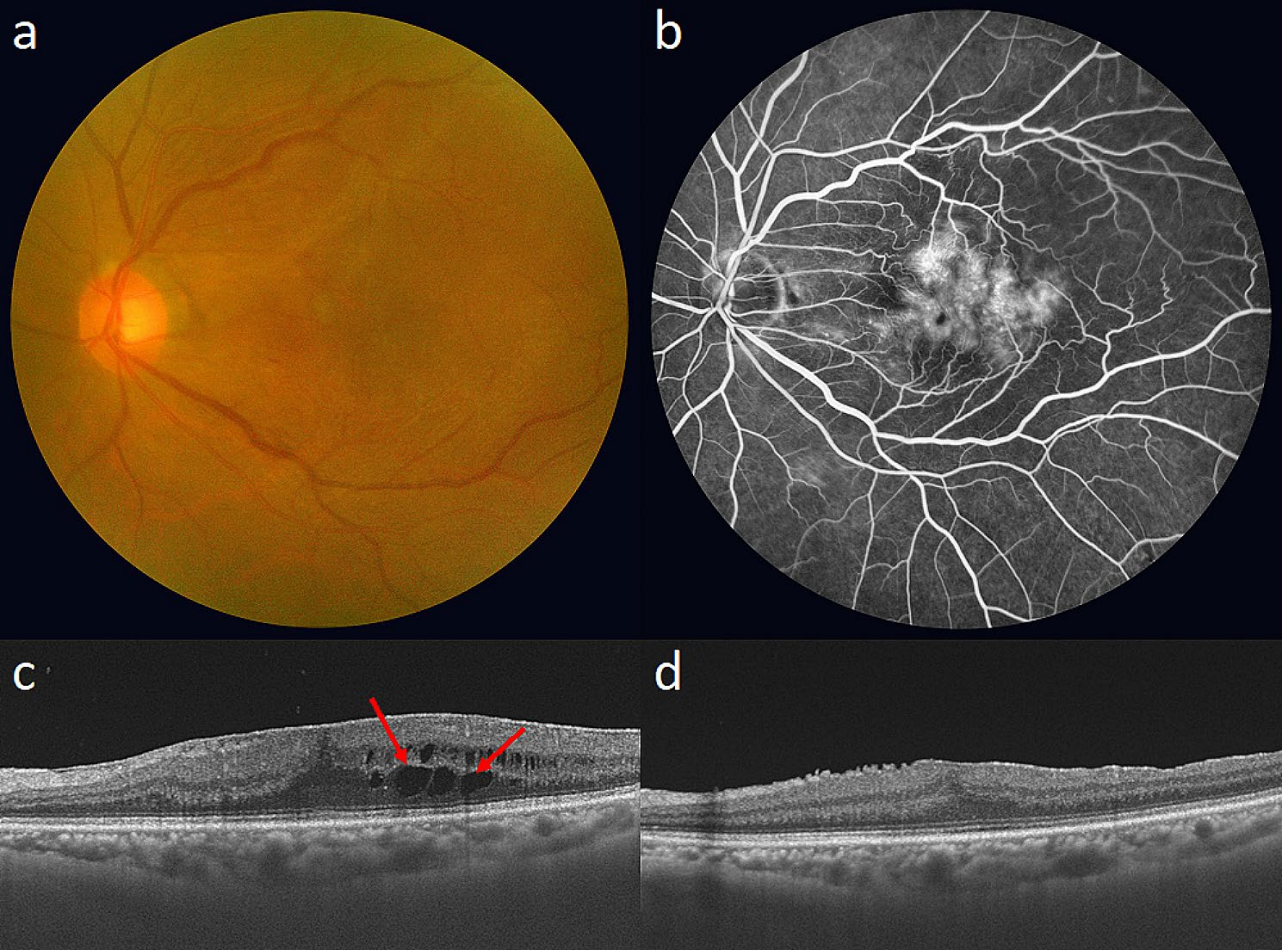
Representative case of a patient not treated with topical bromfenac. (**a**) Fundus photography showed an opaque ERM with retinal folding on macula. (**b**) FA presented leakage on macular centre. (**c**) The IRCs (red arrows) were detected on the baseline OCT. (**d**) The IRCs (arrows) disappeared at 6 months. The BCVA (from 20/40 to 20/30), and CMT (from 499 μm to 367 μm) improved. *BCVA* best-corrected visual acuity, *CMT* central macular thickness, *ERM* epiretinal membrane, *FA* fluorescein angiography, *IRC* intraretinal cystic lesion, *OCT* optical coherence tomography

Of the 63 eyes with ML, 18 eyes (28.6%) were subjected to topical bromfenac (Group A). The changes in the BCVA (0.30 [0.20, 0.40] vs. 0.20 [0.10, 0.30]) and CMT (78.9 ± 61.4 μm vs. 107.2 ± 74.9 μm) did not differ between Group A and B. In aspect of IRC resolution (36.4% vs. 52.6%) and development (14.3% vs. 26.9%), there was no statistical difference between two groups (Fig. 2a). The representative cases of the patients treated with and without topical bromfenac are shown in Figs. 3 and 4. Among 55 eyes without ML, 11 eyes (20.0%) were treated with topical bromfenac (Group A). The changes in the BCVA (0.20 [0.10, 0.30] vs. 0.20 [0.10, 0.30]) and CMT (51.9 ± 64.6 μm vs. 68.2 ± 64.0 μm) did not differ between two groups. In aspect of IRC resolution (50.0% vs. 52.9%) and development (28.6% vs. 18.5%), there was no statistical difference between two groups in eyes without ML (Fig. 2b).

## Discussion

This study highlighted that topical bromfenac did not provide additional benefit in the improvement of the visual and anatomical outcomes following simultaneous cataract and ERM surgery. The BCVA and CMT significantly improved in both eyes with and without topical bromfenac treatment, which did not differ between two groups. The prevention of newly developed IRCs and resolution of pre-existed IRCs were not influenced by topical bromfenac. These findings were consistently observed in eyes with and without ML.

Eyes with iERM present thickened and wrinkled macula with an opaque membrane. Vitrectomy and ERM removal are recommended for the patients with visual impairment. In the present study, we conducted concurrent cataract and ERM removal in all eyes. Additional ILM peeling was also performed. Nearly half of the eyes with iERM demonstrated CME at the baseline, which was observed as IRC on OCT. Mylonas et al. [2] demonstrated that CME develops more often in eyes with previous ERM and ILM removal following cataract surgery. Notably, 5/19 eyes (26%) showed significant morphological changes as CME with cystic formation and/or subretinal fluid on OCT but none of the controls did (*n* = 20) [2]. The authors assumed that eyes with previous ERM surgery were vulnerable to inflammatory mediators inducing CME following cataract surgery. Therefore, there was a concern about that the eyes with concurrent cataract and ERM surgery would be more susceptible to those.

Prostaglandin production in the eyes is the main cause of inflammation-inducing iritis, miosis, hyperaemia, CME, and consequently vision loss after intraocular surgery [13, 15]. Prostaglandins are converted from arachidonic acid via the cyclooxygenase (COX) pathway. Intraocular surgery can be a noxious stimulus to the eye, facilitating the expression of COX-2 [16]. NSAIDs inhibit the synthesis of prostaglandins by irreversibly interfering with the action of both COX-1 and COX-2 [17]. Several studies have reported that topical NSAIDs can effectively lower the concentration of intravitreal PGE2 levels [9, 10, 18]. Topical NSAIDs are clinically preferred for controlling intraocular inflammation, including postoperative CME, particularly before and after cataract surgery [7, 19–21]. Meanwhile, the CME reportedly decreased in most eyes but persists or newly develops in some eyes after ERM surgery [5]. Our study also showed that there was no greater reduction or prevention of CME (e.g., IRC and macular thickness) in eyes treated with topical NSAIDs than in those without it.

There were a few studies to evaluate the effects of topical NSAIDs on ERM surgery. Schoenberger et al. [12] (they excluded cases with concurrent cataract surgery) reported that topical nepafenac elicited faster improvement in the macular thickness, whereas Mandelcorn et al. [22] (they did combined cataract and ERM surgery in 11.3% of subjects) did not confirm this. The final visual acuity and macular thickness did not differ between the eyes with and without topical NSAIDs use in either study. We also observed no beneficial effect on the final anatomical and visual recovery in eyes with topical bromfenac when combined cataract and ERM surgery. The previous and our findings may imply that thorough elimination of tangential traction from membrane is most significant factor and topical NSAIDs is not helpful in improving surgical outcomes further.

IRC on OCT was detected in 43.2% and ML on FA in 53.4% in the current study. They seemed to be not associated with each other, which was similar with previous studies [3, 23]. The IRC, which was also known as intraretinal fluid, CME, microcyst, and schisis cavity [3, 14, 17, 18, 23, 24], could be caused by stretched retinal tissues, degenerative lesions, or actual fluid accumulation from the damaged retinal vessels in the eyes with liERM [4]. Although such features could not be differentiated by FA and OCT, IRCs may not be derived from the ML due to increased inflammatory cytokines. This was supported by previous study that intravitreal concentrations of vasoactive and inflammatory cytokines were similar in ERM with IRC compared healthy controls [24]. This hypothesis also explains the anti-inflammatory drugs including topical NSAIDs and steroid were not beneficial to reduce the IRC and CME in iERM [24, 25]. Meanwhile, in aspect of prevention of newly developed IRC, we expected that topical NSAIDs would have different effects on eyes with and without ML, in particular concurrent cataract and ERM surgery. Because eyes with ML could be more vulnerable to postoperative CME induced by increased cytokines from the damaged retinal microvasculature following intraocular surgery. However, topical bromfenac did not influence the outcomes in both eyes with and without ML. This finding can be also explained by clinical significance of complete removal of traction from membrane in iERM eyes.

This study has several limitations. First, study was retrospectively conducted, and patients were assigned to each group by the year due to application of topical bromfenac. Time difference might affect the surgical outcome although all surgeries were performed by an experienced surgeon. Secondly, we only conducted FA at the baseline. If follow-up FA was performed, the changes in CME could have been attributed to actual angiographic CME (Irvine-Gass syndrome) or other types. Third, topical steroids were used postoperatively for all eyes. This might have minimalized the effects of the topical NSAID on the surgical outcomes. However, the 2-week application of topical steroids was a relatively short period to treat and prevent postoperative CME during the 6 months follow-up period of this study. Forth, we just evaluated the effect of topical bromfenac on the eyes with iERM. If different drug were applied on different diseases, different outcomes would be found. Finally, we did assess the existence and disappearance of the IRC in this study. If the quantitative analysis for IRCs was conducted, more detailed changes would be found; therefore, future studies are recommended to analyse this aspect.

Topical bromfenac did not show any additional benefits in final visual and anatomical outcomes following concurrent cataract and iERM surgery in eyes with ML and also in those without ML. Only in eyes which achieved final IRC resolution, topical bromfenac seemed to help in faster resolution (of 25 eyes with final IRC resolution, 6/6 (100%) in group A vs. 8/19 eyes (42.1%) in group B achieved IRC resolution at 3 months). Complete removal of the tractional force from epiretinal membrane is the principal element to determine outcomes of iERM surgery, which could have overpowered the additional effect of topical bromfenac in this study. However, because of the limitations of our study described above, the effect of topical bromfenac could be inconclusive. Larger randomised prospective studies are warranted to establish this effect further.

## Data Availability

The data that support the findings of this study are available from the corresponding author.

## Abbreviations

BCVA: Best-corrected visual acuity
CME: Cystoid macular edema
CMT: Central macular thickness
EIFL: Ectopic inner foveal layer
iERM: Idiopathic epiretinal membrane
FA: Fluorescein angiography
ILM: Internal limiting membrane
IRC: Intraretinal cystic lesion
ML: Microvascular leakage
OCT: Optical coherence tomography
